# An artificial intelligence approach for predicting death or organ failure after hospitalization for COVID-19: development of a novel risk prediction tool and comparisons with ISARIC-4C, CURB-65, qSOFA, and MEWS scoring systems

**DOI:** 10.1186/s12931-023-02386-6

**Published:** 2023-03-13

**Authors:** Stephen Wai Hang Kwok, Guanjin Wang, Ferdous Sohel, Kianoush B. Kashani, Ye Zhu, Zhen Wang, Eduardo Antpack, Kanika Khandelwal, Sandeep R. Pagali, Sanjeev Nanda, Ahmed D. Abdalrhim, Umesh M. Sharma, Sumit Bhagra, Sagar Dugani, Paul Y. Takahashi, Mohammad H. Murad, Mohammed Yousufuddin

**Affiliations:** 1 Harry Butler Institute, Murdoch University, Murdoch, Australia; 2 Department of Information Technology, Murdoch University, Murdoch, Australia; 3 Division of Nephrology and Hypertension, Mayo Clinic, Rochester, MN USA; 4 Robert D. and Patricia E. Kern Centre for the Science of Healthcare Delivery, Mayo Clinic, Rochester, MN USA; 5 Division of Hospital Internal Medicine, Mayo Clinic Health System, Austin, MN USA; 6 Division of Surgery, Mayo Clinic, Rochester, MN USA; 7 Division of Hospital Internal Medicine, Mayo Clinic, Rochester, MN USA; 8 Division of General Internal Medicine, Mayo Clinic, Rochester, MN USA; 9 Division of Hospital Internal Medicine, Mayo Clinic, Phoenix, AZ USA; 10 Department of Endocrine and Metabolism, Mayo Clinic Health System, Austin, MN USA; 11 Division of Community Internal Medicine, Mayo Clinic, Rochester, MN USA; 12 Division of Preventive Medicine, Mayo Clinic, Rochester, MN USA; 13 Hospital Internal Medicine, Mayo Clinic Health System, Mayo Clinic, 1000 1st Drive NW, Austin, MN USA

**Keywords:** COVID-19, Mortality, Organ failure, Prediction models, Machine learning algorithms

## Abstract

**Background:**

We applied machine learning (ML) algorithms to generate a risk prediction tool [Collaboration for Risk Evaluation in COVID-19 (CORE-COVID-19)] for predicting the composite of 30-day endotracheal intubation, intravenous administration of vasopressors, or death after COVID-19 hospitalization and compared it with the existing risk scores.

**Methods:**

This is a retrospective study of adults hospitalized with COVID-19 from March 2020 to February 2021. Patients, each with 92 variables, and one composite outcome underwent feature selection process to identify the most predictive variables. Selected variables were modeled to build four ML algorithms (artificial neural network, support vector machine, gradient boosting machine, and Logistic regression) and an ensemble model to generate a CORE-COVID-19 model to predict the composite outcome and compared with existing risk prediction scores. The net benefit for clinical use of each model was assessed by decision curve analysis.

**Results:**

Of 1796 patients, 278 (15%) patients reached primary outcome. Six most predictive features were identified. Four ML algorithms achieved comparable discrimination (*P* > 0.827) with c-statistics ranged 0.849–0.856, calibration slopes 0.911–1.173, and Hosmer–Lemeshow *P* > 0.141 in validation dataset. These 6-variable fitted CORE-COVID-19 model revealed a c-statistic of 0.880, which was significantly (P < 0.04) higher than ISARIC-4C (0.751), CURB-65 (0.735), qSOFA (0.676), and MEWS (0.674) for outcome prediction. The net benefit of the CORE-COVID-19 model was greater than that of the existing risk scores.

**Conclusion:**

The CORE-COVID-19 model accurately assigned 88% of patients who potentially progressed to 30-day composite events and revealed improved performance over existing risk scores, indicating its potential utility in clinical practice.

**Supplementary Information:**

The online version contains supplementary material available at 10.1186/s12931-023-02386-6.

## Background

COVID-19 continues to disrupt healthcare systems with unacceptably high hospitalization and death rates in the United States. The Centers for Disease Control and Prevention’s COVID data tracker weekly review reported current 7-day average of 4216 new hospitalizations and new 537 deaths as of January 25, 2023 [1]. The risk of progression to critical organ dysfunction or death varies considerably among patients hospitalized for COVID-19 vary considerably with estimates ranging from 3 to 80% [2]. A substantial proportion of patients with mild to moderate symptoms on admission may rapidly progress to critical illness [3], necessitating prompt attention to choose the best possible forward strategy. Therefore, the early identification of patients at the greatest risk for unfavorable outcomes with COVID-19 is crucial for clinical decision-making and resource allocation.

Several promising prognostic models and risk-scoring systems, mainly using standard statistical (SS) approaches have been developed to predict COVID-19 outcomes. A systematic review identified 39 prediction models based on SS methods for predicting short-term COVID-19 outcomes [4, 5]. However, most studies using these models have serious methodological flaws and a high risk of bias in multiple domains. Numerous machine learning (ML) models have also been developed using a priori or large heterogeneous electronic health record (EHR) data in patients with COVID-19. Although the results were promising for the diagnosis, they were inconclusive regarding outcome prediction after COVID-19. None of the available prognostic models has sufficient clinical utility to inform clinical decision-making in hospitalized patients with COVID-19.

Accordingly, we conducted a retrospective multicenter cohort study to develop robust multivariable ML models to identify a set of most predictive variables to generate a point-based new risk prediction tool [Collaboration for Risk Evaluation in COVID-19 (CORE-COVID-19)] that can be used at the bedside to predict a composite of endotracheal intubation, intravenous vasopressor administration, or death within 30 days of admission for COVID-19. We extended our objectives to compare the ML models and CORE-COVID-19 model with previously identified and validated risk prediction tools for COVID-19 outcomes.

## Methods

Additional details of methods are provided in Additional file 1: Panel 1. Methods, additional description.

### Data source

Data were extracted from the Mayo Clinic’s comprehensive electronic health record system encompassing all 16 Mayo Clinic hospitals across four states (Arizona, Florida, Minnesota, and Wisconsin) from March 2020 to February 2021. We used *International Classification of Disease, Tenth Revision, Clinical Modification* (ICD-10-CM) codes U07.1, J12.89, J12.82, J20.8, J40, J22, J98.8, or J80 for data extraction [6]. These ICD-10-CM COVID-19 diagnosis codes were shown to reliably capture COVID-19 discharges with sensitivity, specificity, positive predictive value, and the negative predictive value of 98.01%, 99.04%, 91.52%, and 99.79%, respectively [7]. Additionally, we used “Mayo Data Explorer (MDE)”, a Mayo Clinic-specific server, to identify patients using the term “COVID-19” to extract COVID-19 patient data to supplement the initial ICD-10-CM codes-derived data. The use of two different servers for the extraction of COVID-19 patients potentially minimizes missing COVID-19 patients. Finally, we conducted a manual review of the electronic medical records of each patient to verify the accuracy of the data and add the missing data points.

### Study design and population

This was a retrospective study of consecutive adults hospitalized with reverse transcription-polymerase chain reaction-confirmed COVID-19. The investigators reviewed the discharge diagnoses of COVID-19. Pregnant patients and those who declined access to their medical records for research were excluded. Details of the process of data extraction were published previously [8]. Data were de-identified according to the United States Department of Health and Human Services privacy rules [9] before analysis. The study conformed to the Declaration of Helsinki, strengthening the reporting of observational studies in epidemiology (STROBE) statement [10], and the transparent reporting of a multivariable prediction model for individual prognosis or diagnosis (TRIPOD) reporting guidelines [11]. The Mayo Clinic Institutional Review Board approved the study and waived the need for informed consent.

### Variable selection

The inclusion of independent variables for model development was based on a comprehensive review of relevant prognostic studies in patients with COVID-19 [4, 5, 12–16], non-COVID-19 pneumonia [17–22], and expert opinion. Heart rate, respiratory rate, systolic blood pressure, diastolic blood pressure, temperature, and SpO_2_ (oxygen saturation) were time-varying dynamic variables. For each dynamic variable, we ascertained an average of the three consecutive measurements obtained at 15 min intervals on admission for analysis. The variable selection was performed to eliminate potentially unrelated variables and enhance the prediction model’s performance [23]. We identified 92 potential predictor variables for model development including those related to demographics (n = 3), social indicators (n = 4), anthropometric measure (n = 1), admission source (n = 4), admitting service (n = 3), comorbid conditions (n = 31), vital (8), laboratory measures (n = 16), ECG measure (n = 1), hospital complications (n = 12), and drugs (n = 9). Hypotension as an input feature was defined as systolic blood pressure < 90 mmHg that responded to fluid bolus or medication adjustment. Other key complications which were noted during hospitalizations and included as input features were encephalopathy, hypothermia, pulmonary edema, myocardial infarction, pulmonary embolism, and respiratory failure that preceded the progression to composite events were also included as input features. These incidents occurred prior to progression to the composite events.

### Data pre-processing

The missing values for continuous variables were imputed by the bagged trees method and dichotomous variables by the mode value [24, 25]. The continuous variables were further transformed by the Yeo-Johnson transformation to reduce skewness, and then centered and scaled. The categorical variables, i.e., the SpO_2_ categories were converted to dummy variables by one-hot encoding, so the number of input features increased from 92 to 98, plus one outcome label variable. Finally, the pre-processed data were randomly split [26] into training (70%) and validation (30%) sets for model development and internal validation.

### Data-driven feature selection

A data-driven feature selection process was implemented on development set after data pre-processing. We incorporated Recursive Feature Elimination (RFE) method [27, 28], which is a backward feature selection algorithm. It can fit ML classifiers such as logistic regression (LR), Naïve Bayes (NB), and Random Forest (RF) in our study, to select a subset of variables important in predicting the outcome. Previous studies demonstrated good capability of RFE in enhancing the prediction performances of the three classifiers [29–31]. These classifiers are familiar for working with RFE to generate reliable results. In the RFE procedure, number of variables ranging from 2 to 92 were retained in the model and the variable set with the best accuracy in predicting the outcome was identified. The procedure was completed with tenfold cross-validation and repeated five-times. The RFE procedure for each classifier was performed 30 times on different seeds; thus, there were 90 best accuracies to compare. As a result, the six features selected by the logistic regression in RFE demonstrated the best accuracy considering a small number of features required. We calculated the level of importance of the variables in the selected model [32]. Finally, the six selected variables were used for the subsequent stages of model development.

### Analytic approach (Fig. 1)

**Fig. 1:**
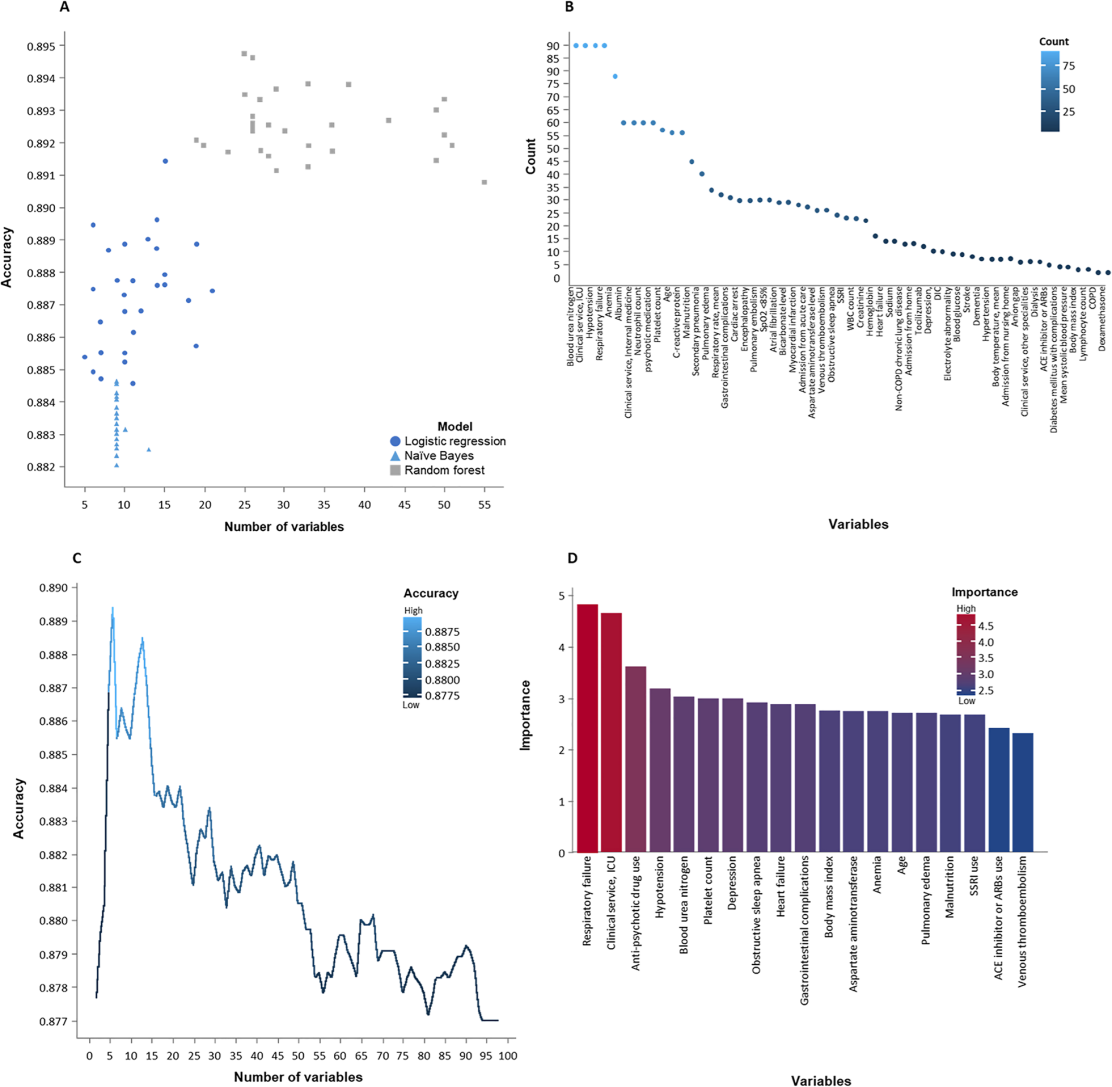
Schematics of data processing. **A** shows selected models with variable sets of the highest accuracies in ninety RFE procedures; the models involved in the RFE procedures were logistic regression, Naïve Bayes, and random forest; **B** illustrates number of times a variable was selected among the ninety RFE procedures; the count was the frequency for a feature to be chosen among the RFE procedures; **C** numbers of variables retained and tested in the RFE procedure in which the final chosen model was generated; the accuracy was the ratio of the number of correct predictions to the total number of predictions; **D** The variable importance level of the chosen model concerning the first nineteen features; the importance was the scaled score of the variable importance for the linear model. Abbreviations. ACE, angiotensin converting enzyme; ICU, intensive care unit; SSRI, selective serotonin receptor inhibitors

*ML-based models* We reviewed the literature through March 2022 to identify potential ML models used to predict disease prognosis among patients with COVID-19 [4, 33]. Based on the study sample size, volume and complexity of the data, we constructed artificial neural network (NN) [34], support vector machine (SVM) [35], gradient boosting (GBM) [36], and LR. The LR was considered the reference model since LR was one of the most common methods used in health research and clinical analysis. The data subset of the six variables and the outcome label were used to train and test SVM, GBM, NN and the LR classifier. The description of machine learning models is provided in Additional file 1: Table S1 Machine learning models.

*Model development and parameter tuning* Each ML model was trained with parameter (hyperparameter) tuning to define the model architecture [37, 38]. The tune length was set to accommodate a range of random values of the tuning parameter or the unique combination of values if there were more than one tuning parameter for a ML model. Therefore, a range of candidate values were tested to determine the best tuning value or combination of values for optimal model architecture. Each ML algorithm was tuned via a tune length of 300 candidate parameter values or parameter value combination, with tenfold cross validation and repeated five times.

The parameters were referred to the tuning parameters of the ML models, which help define the model architecture in the training process [37]. The values of tuning parameter(s) need to be pre-determined to construct ML models. One or a few tuning parameters need to be set in a ML algorithm in classifier training. Tune length is the total number of unique parameter values, or unique combinations of parameter values if there is more than one parameter, required for a ML model in the training process. For instance, the 300 candidate parameter values or combinations of parameter values concerning a respective ML model is the range of candidate values to be tested to determine the optimal model architecture. Each algorithm was tuned via a random grid search from 300 candidate parameters or parameter combinations, with tenfold cross-validation repeated five times [39, 40].

*Ensemble model (EM)* We combined the results of the NN, SVM, and GBM models to generate an ensemble ML model, which is a single ML model that combines multiple classification models using linear regression [41].

### Development of a point-based CORE-COVID-19 model

The six selected variable and an outcome label were used to develop the COR-COVID-19 point-based scoring system using Xie and colleagues [42] method to risk-stratify patients for the composite outcome. Six variables and outcome label were used to train and test the four ML models i.e., SVM, GBM, NN and the LR classifier, here the LR ML model is the “reference model” i.e., baseline model. The training data of the six variables and outcome label were used to develop the CORE-COVID-19 model, using LR technique in score weighting. The testing data were used for validation. The data subset of the six variables and outcome label were used to run a simple LR, generating the estimates and odds ratio. Continuous independent variables were converted into categorical variables based on five quantiles: 0.05, 0.2, 0.8, 0.95, and 1 [43]. The score weighting for each variable category was performed by LR. The cutoff values of the continuous variables were fine-tuned based on the first weighting results. Performance metrics were obtained from the validation dataset after fine-tuning. The total score was set to 16 for easy manual calculations.

### Validation and evaluation of existing risk-prediction tools

Through an updated search to June 2022, we found that “International Severe Acute Respiratory and emerging Infections Consortium Coronavirus Clinical Characterization Consortium (ISARIC-4C)” model [44], “Confusion, Urea, Respiratory rate, Blood pressure, and age ≥ 65 years (CURB-65) [19]”, “quick Sequential Organ Failure Assessment (qSOFA)” [45], and “Modified Early Warning Score (MEWS)” [46] were the most feasible for validation and recalibration. The ISARIC-4C was originally developed in a hospitalized COVID-19 population in the United Kingdom and was identified as the most promising prediction model for COVID-19 outcome prediction [4, 5]. Although CURB-65, qSOFA, and MEWS were developed for the non-COVID-19 population, they share similar characteristics, and their prognostic implications in COVID-19 have recently been explored. In the present study, ISARIC-4C, CURB-65, qSOFA, and MEWS scores were calculated for each patient. The dichotomous Glasgow Coma Scale (15 vs. < 15) was replaced by the presence or absence of metabolic encephalopathy. The unit of blood urea nitrogen (BUN) in mg/dl was multiplied by a conversion factor of 0.3571 to convert to mmol/L for estimating the ISARIC-4C score [47]. ISARIC-4C, CURB-65, qSOFA, and MEWS scores were validated and recalibrated. A brief description of the existing risk prediction tool identified for external validation in Additional file 1: Table S2 Description of existing risk prediction tools, and Table S3 Risk prediction models and estimated scores in Additional file 1.

### Outcome

The outcome was a composite of endotracheal intubation, intravenous vasopressor administration, or death from any cause within 30-days of hospitalization for COVID-19, whichever occurred first.

### Statistical analysis

*General.* We reported the mean and standard deviation (SD) for normally distributed variables, the median and interquartile range (IQR) for non-normally distributed variables, and the number and proportion for categorical variables. Univariate analyses were performed using the Student *t* test, Kruskal–Wallis test, and Pearson *χ*^2^ test for univariate analyses as appropriate. Statistical significance was adjusted to P < 0.0005 to account for multiple comparisons using Bonferroni’s method.

*Standard performance metrics.* The ML models’ performances were evaluated in the development and validation datasets, whereas the CORE-COVID-19, ISARIC-4C, CURB-65, qSOFA, and MEWS were assessed in the cumulative cohort. Receiver operating characteristic (ROC) curves were generated for each model. Discrimination was quantified using the area under the ROC curves (AUC). To account for outcome prevalence, we reported the sensitivity, specificity, positive predictive value (PPV), negative predictive value (NPV), and accuracy. The model performance was rated using the F1 score and Kappa statistics. Performance metrics were compared using Kruskal–Wallis test across the models and the Hosmer–Lemeshow test for goodness-of-fit [48].

*Calibration.* The agreement between the probability of prediction and actual observation was estimated for each model [49]. For each model, calibration performance was assessed using the Brier score, Hosmer–Lemeshow test, and calibration plots.

*Decision curve analysis (DCA)*. We performed DCA to determine the model’s net benefit relative to harm in predicting the composite outcome [50]. DCA accounts for the tradeoff between harms and benefits across a range of thresholds associated with the use of the risk prediction model to ascertain whether or not to risk stratify the patients using the model [51]. In this study, the terms “treat all” and “treat none were replaced by “intervention for all” and “intervention for none,” respectively. These terms are more appropriate in context of the present study and as recommended by Vickers et al. [52].

*Analysis of the CORE-COVID-19 model.* LR analysis was conducted to regress the study outcome on the selected variables to compute estimates and odds ratios (ORs). The CORE-COVID-19 total scores were stratified into tertiles of equal size to support clinical use and compared using Kaplan–Meier method and Cox regression models.

## Results

### Study population

Additional file 1: Figure S1 illustrates the STROBE flow diagram for patient selection. A total of 3845 patient hospitalized for COVID-19 were initially identified from the Mayo Clinic database. Data analysis was performed in 1800 randomly selected patients owing to restriction on larger data sharing for patient privacy. The study cohort of 1800 patients were comparable to the remaining 2045 patients of the initial cohort. Four patients were excluded due to incomplete outcome data. The final study cohort comprised 1796 adults with a median age of 68 years (range 18–89 years), 42% women, and 83% whites. The development cohort and validation cohort were comparable in all measured characteristics (Additional file 1: Table S4. Characteristics of study population by the development and validation cohorts) whereas patients who progressed to composite outcomes significantly differed in multiple domains from those who did not (Table 1). The proportion of patients who experienced the composite outcome was similar across the participating states (*P* = 0.683). At a median of 8-days (IQR 3, 13), 96 patients (5.4%) were intubated for respiratory failure, 63 patients (3.5%) received intravenous vasopressors for circulatory failure, and 119 patients (6.6%) died. The 30-day composite of death or critical organ failure requiring life support was observed in 278 (15.5%) patients. The median length of hospital stay was 6 days (IQR 4, 10).

**Table 1:** Characteristics of study population by composite outcome

	Patients with no composite outcome, n = 1518	Patients with composite outcome, n = 278	*P* value*
Demographics			
Age, years (standard deviation)	65 (15)	71 (13)	< 0.0001
Female, n = (%)	654 (43)	102 (37)	0.0472
White, n = (%)	1266 (84)	221 (79)	0.1129
Social indicators			
Married, n = (%)	919 (60)	156 (56)	0.1664
Current smoker, n = (%)	73 (5)	13 (5)	0.9241
Ever smoker, n = (%)	573 (38)	123 (44)	0.0409
Substance use disorder, n = (%)	48 (3)	12 (4)	0.3247
Anthropometric measure			
Body mass index, kg/m^2 ^	31 (8)	30 (7)	0.0035
Admission source			
Home, n = (%)	1264 (83)	171 (61)	< 0.0001
Clinic, n = (%)	56 (4)	9 (3)	0.7109
Acute care, n = (%)	126 (8)	70 (25)	< 0.0001
Nursing home, n = (%)	72 (5)	28 (10)	0.0004
Admitting service			
Intensive care unit, n = (%)	74 (5)	113 (40)	< 0.0001
Intern medicine, n = (%)	1009 (66)	100 (36)	< 0.0001
Other service, n = (%)	435 (29)	65 (23)	0.0712
Comorbid conditions			
Anemia, n = (%)	306 (20)	122 (44)	< 0.0001
Arthritis, n = (%)	164 (11)	41 (15)	0.0572
Atrial fibrillation, n = (%)	301 (20)	103 (37)	< 0.0001
Asthma, n = (%)	108 (7)	15 (5)	0.2669
Bone marrow disease, n = (%)	74 (5)	22 (8)	0.0384
Bone marrow/stem cell transplant, n = (%)	14 (1)	2 (0.7)	0.7407
Coronary artery disease, n = (%)	126 (8)	38 (14)	0.0043
Cancer, active, n = (%)	80 (5)	24 (9)	0.0273
Cancer with metastasis, n = (%)	36 (2)	5 (2)	0.5565
Chronic kidney disease, n = (%)	331 (22)	80 (29)	0.0110
Chronic obstructive pulmonary disease, n = (%)	215 (14)	60 (22)	0.0016
Depression, n = (%)	228 (19)	53 (19)	0.9712
Dementia, n = (%)	29 (2)	13 (5)	0.0050
Diabetes mellitus, n = (%)	556 (37)	134 (48)	0.0003
Diabetes with complications, n = (%)	321 (21)	73 (26)	0.0582
Heart failure, n = (%)	223 (15)	72 (26)	< 0.0001
Human immuno-deficiency virus, n = (%)	2 (0.1)	1 (0.3)	0.3922
Hyperlipidemia, n = (%)	770 (51)	168 (60)	0.0029
Hypertension, n = (%)	971 (64)	223 (80)	< 0.0001
Immunodeficiency, n = (%)	66 (4)	20 (7)	0.0410
Liver disease, n = (%)	36 (2)	14 (5)	0.0130
Malnutrition, n = (%)	102 (7)	50 (18)	< 0.0001
Obstructive sleep apnea, n = (%)	371 (24)	54 (19)	0.0705
Osteoporosis, n = (%)	98 (6)	22 (8)	0.3708
Other lung conditions, n = (%)	66 (4)	34 (12)	< 0.0001
Other neurological conditions, n = (%)	51 (3)	12 (4)	0.4253
Other psychiatric conditions, n = (%)	128 (8)	9 (3)	0.0027
Peripheral artery disease, n = (%)	6 (0.3)	2 (0.7)	0.4555
Solid organ transplant, n = (%)	71 (5)	14 (5)	0.7954
Stroke, n = (%)	54 (4)	28 (10)	< 0.0001
Venous thrombo-embolism, n = (%)	143 (9)	54 (19)	< 0.0001
Vitals			
Heart rate/min, (standard deviation)	89 (18)	93 (20)	0.0077
Respiratory rate/min, (standard deviation)	22 (5)	25 (8)	< 0.0001
Systolic blood pressure, mmHg (standard deviation)	131 (19)	126 (21)	< 0.0001
Diastolic blood pressure, mmHg (standard deviation)	77 (12)	73 (13)	< 0.0001
Temperature in celcius (standard deviation)	37.1 (0.6)	37.2 (0.7)	0.6469
SpO_2_, 94%–100%, n = (%)	970 (64)	127 (46)	< 0.0001
90–93%, n = (%)	450 (30)	83 (30)	0.9433
< 90%, n = (%)	98 (6)	68 (24)	< 0.0001
Laboratory measures			
Hemoglobin, g/dL (standard deviation)	12.9 (2.0)	12.4 (2.3)	< 0.0001
White blood cell count, ×10 (interquartile range)	6.3 (4.6, 8.5)	8.2 (5.3, 12.4)	< 0.0001
Neutrophils, ×10 (interquartile range)	4.7 (3.2, 6.6)	6.6 (4.2, 10.6)	< 0.0001
Lymphocytes, x10 (interquartile range)	0.8 (0.6, 1.16)	0.8 (0.5, 1.1)	0.0001
Platelets, ×10 (interquartile range)	209 (86)	198 (93)	0.0503
Sodium, mmol/L (standard deviation)	135 (4.5)	135 (6)	0.2301
Bicarbonate, mmol/L (standard deviation)	23.7 (3.5)	22.1 (4.3)	< 0.0001
Anion gap, mmol/L (standard deviation)	12.4 (2.9)	13.4 (3.5)	< 0.0001
Blood urea nitrogen, mg/dL (interquartile range)	17 (12, 25)	28 (20, 41)	< 0.0001
Creatinine, mg/dL (interquartile range)	1.0 (0.8, 1.3)	1.1 (0.9, 1.7)	< 0.0001
Albumin, g/dL (standard deviation)	3.6 (0.5)	3.3 (0.6)	< 0.0001
Alanine aminotransferase, U/L (interquartile range)	28 (19, 46)	29 (19, 49)	0.4232
Aspartate aminotransferase, U/L (interquartile range)	38 (29, 55)	47 (32, 71.3)	< 0.0001
Bilirubin, mg/dL (interquartile range)	0.6 (0.3, 0.9)	0.5 (0.3, 0.7)	0.0038
Glucose, mg/dL (standard deviation)	137 (54)	154 (71)	< 0.0001
c-reactive protein, mg/L (interquartile range)	61.0 (24.7, 113.2)	104.7 (19.4, 362.6)	< 0.0001
ECG measure			
QTc, msec (standard deviation)	449 (30)	455 (35)	0.0029
In-hospital complications			
Hypothermia, n = (%)	7 (0.5)	8 (3)	< 0.0001
Hypotension, n = (%)	155 (10)	124 (45)	< 0.0001
Myocardial infarction, n = (%)	13 (0.8)	17 (6)	< 0.0001
Cardiac arrest, n = (%)	2 (0.1)	20 (7)	< 0.0001
Respiratory failure, n = (%)	755 (50)	259 (93)	
Pulmonary edema, n = (%)	16 (1)	24 (9)	< 0.0001
Pulmonary embolism, n = (%)	91 (6)	61 (22)	< 0.0001
Secondary pneumonia, n = (%)	145 (10)	91 (33)	< 0.0001
GI complications, n = (%)	20 91)	36 (13)	< 0.0001
Electrolyte abnormality, n = (%)	890 (59)	230 (83)	< 0.0001
Disseminated intra-vascular coagulation, n = (%)	2 (0.1)	6 (2)	< 0.0001
Encephalopathy, n = (%)	62 (0.4)	66 (24)	< 0.0001
Drugs			
Remdesivir, n = (%)	162 (11)	45 (16)	0.0081
Tocilizumab, n = (%)	15 (1)	15 (5)	< 0.0001
Dexamethasone, n = (%)	338 (22)	83 (30)	0.0060
Hydroxychloroquine, n = (%)	25 (2)	11 (4)	0.0115
Aspirin, n = (%)	534 (35)	111 (40)	0.1291
Statin, n = (%)	564 (37)	105 (38)	0.8452
ACEI/ARBs, n = (%)	426 (28)	60 (22)	0.0254
Anti-psychotic medication, n = (%)	90 (6)	57 (21)	< 0.0001
SSRI, n = (%)	204 (13)	26 (9)	0.0609

Abbreviations: ACEI/ARBs, angiotensin converting enzyme inhibitor/Angiotensin II receptor blockers; SSRI, selective serotonin receptor inhibitor

*P value was significant at < 0.0001 for multiple comparison according to Bonferroni method

### Variable selection (Fig. 1)

Among the RFE procedures, the LR, NB, and RF selected 5–21, 9–13, and 19–55 variables, respectively. The six variables selected by LR in a RFE procedure offered the best accuracy (0.8895) considering a small number of features needed. The levels of importance of the variables were calculated, and six variables were used for the development of four ML models and the point-based CORE-COVID-19 model. The six chosen variables were incident respiratory failure, hypotension, admission to intensive care unit (ICU), BUN, platelet count, and exposure to antipsychotic medication. The respiratory failure was defined as a PaO_2_ ≤ 60 mmHg, SpO_2_ ≤ 90%, PaO_2_/FiO_2_ < 300, and/ or PaCO_2_ ≥ 50 mmHg on ambient air; requiring 4 L/min or more oxygen to maintain SpO_2_ ≥ 92% for a minimum of 2 hours; or requiring at least 2 L/min of oxygen continuously for > 24 h. Hypotension was defined as a systolic blood pressure < 90 mmHg or a mean arterial pressure < 60 mmHg for > 30 min that responded to fluid boluses and/or adjustment of medications before the time to outcome event. For antipsychotic medications, exposure was counted regardless of whether it was a reconciled by home medication list or newly administered in the hospital before the time to the outcome event. Of all the potential predictor variables, admitting service (admission to ICU, internal medicine, or other services) has been a hospital level characteristic. Notably, admission to the ICU could have varied based on the hospital, attending physician, and level of healthcare system strain, and finally contingent on clinician judgment. These events occurred prior to outcome event.

### ML models

**Fig. 2:**
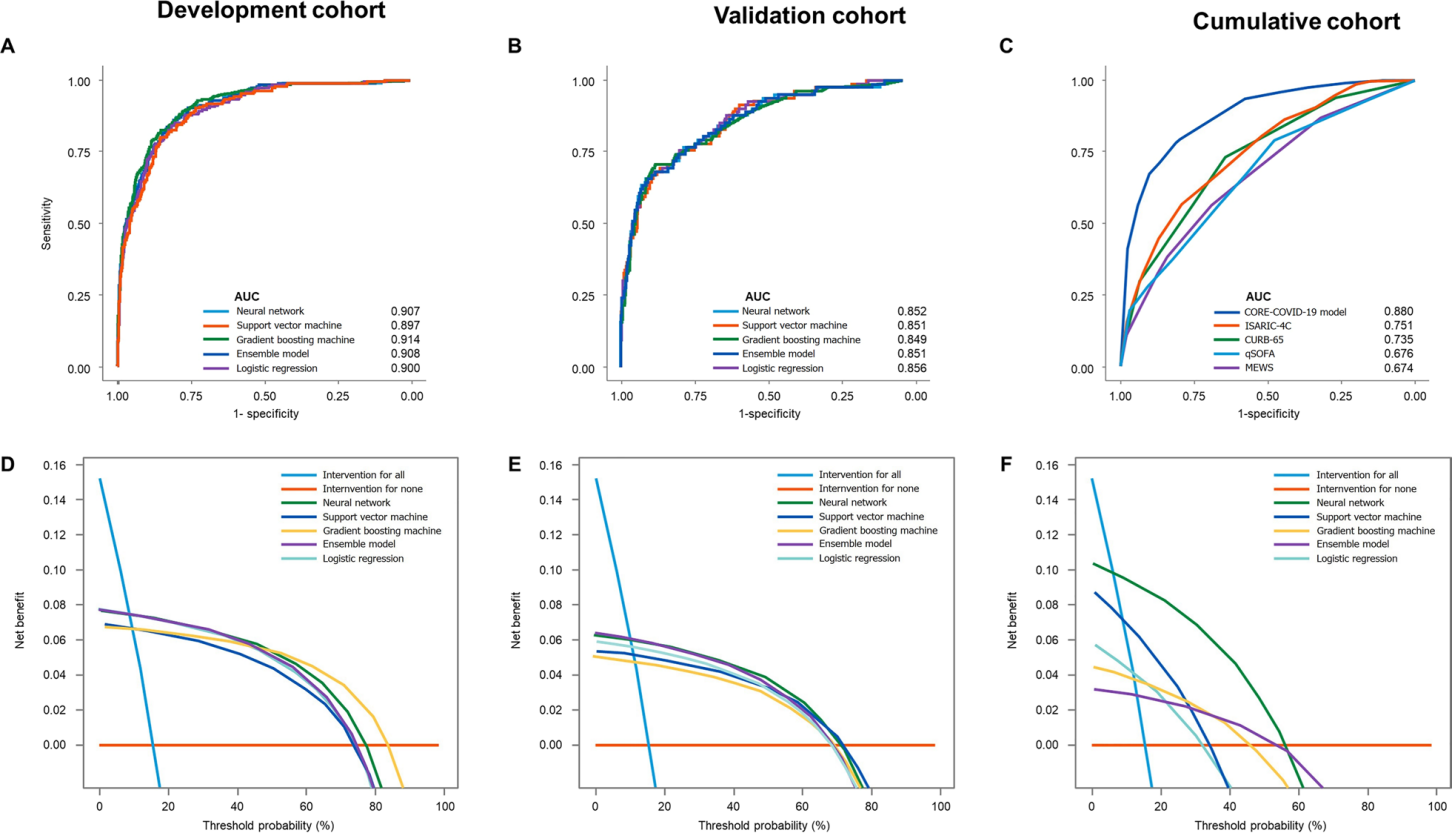
Receiver operating characteristic curves (ROC) for predicting the composite of death or organ failure at 30 days after hospitalization for COVID-19. (**A**) development and (**B**) internal validation datasets stratified according to individual machine learning models; Fig. 2**C** shows ROC for predicting outcome stratified by the new CORE-COVID-19 and 4 existing risk prediction tools. CORE-COVI-19 model consistently outperformed each existing risk prediction tools. Fig. 2**D** and **E** showed decision curve analysis stratified according to machine learning models in development (**D**) and validation (**E**) data sets. Fig. 2**F** illustrate decision curve analysis stratified by CORE-COVID-19 and other existing risk prediction tools for outcome prediction with net benefit of CORE-COVID-19 exceeding that of other models at wide range of thresholds. The "intervention for all" indicated net benefit from 0 to 0.15 below 20% of threshold probability. The ML models achieved the best net benefit at around .07–.08 when the threshold probability approached the minimum in the training dataset. The models still showed net benefit when the threshold probability rose to approximately 75%; the GBM even showed net benefit at above 80% of threshold probability. On the validation data set, the best net benefit ranged between .05–.07, and the models offered net benefit at around 70% of threshold probability at most. The maximum net benefit for CORE-COVID-19 model was best at 0.1 threshold and continued to show net benefit at above 55% of threshold probability which was higher than existing prediction tools. ISARIC-4C had its best net benefit, which was comparable to ML models in training, but the maximum threshold probability showing net benefit was only around 35%. The qSOFA presents net benefit at above 50% of threshold probability but its best net benefit was only approximately 0.03. Abbreviations: AUC, area under receiver operating characteristic curve; CORE-COVID-19, Collaboration for Risk Evaluation in COVID-19; CURB-65, confusion, urea, respiratory rate, blood pressure, and age ≥ 65 years; ISARIC-4C, International Severe Acute Respiratory and emerging Infections Consortium Coronavirus Clinical Characterization Consortium; qSOFA, quick sequential organ failure assessment; MEWS, modified early warning score

**Fig. 3:**
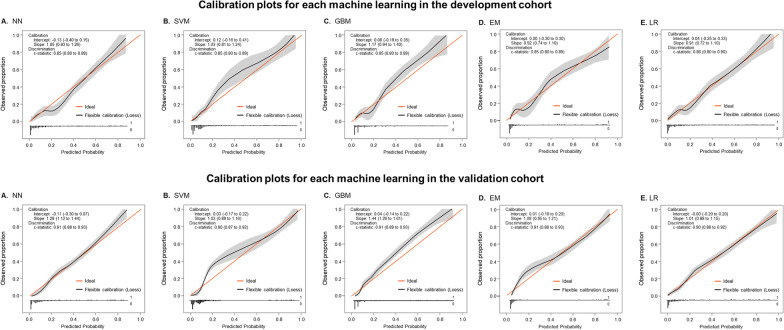
Calibration plots associated with each machine learning model in development (upper panel **A**–**E**) and validation (lower panel, **A**–**E**) datasets, all showed good calibration

The performance metrics were comparable across the ML models and EM in development and validation datasets (Fig. 2A and B; Table 2). The ML models’ AUC, accuracy, F1 score, and Briers scores in the validation dataset were 0.852, 89%, 0.935, and 0.087 for NN; 0.851, 88%, 0.933, and 0.089 for SVM; 0.849, 88%, 0.931, and 0.089 for GBM; 0.856, 88%, 0.932, and 0.0861 for LR; and 0.851, 88%, 0.935, and 0.088 for EM; respectively. Sensitivity, specificity, PPV, NPV, and Kappa values were similar across the models (Table 2). The Hosmer–Lemeshow test revealed *P* > 0.05 for all models in both the development and validation datasets. Figure 3 illustrates calibration plots with intercept, slope, and corresponding 95% confidence intervals (CI) for each model in the development and validation datasets.

**Table 2:** Performance metrics for each model in development, validation, and cumulative cohorts

Dataset	Model	AUC (95% CI)	Sensitivity (95% CI)	Specificity (95% CI)	PPV (95% CI)	NPV (95% CI)	F1 score*	Kappa^†^	Brier score	Cut-off	Youden index**
Development	NN	0.907 (0.885–0.929)	0.973 (0.961–0.982	0.495 (0.423–0.567)	0.913 (0.895–0.928)	0.770 (0.686–0.840)	0.942	0.547	0.078	0.162	0.670
	SVM	0.897 (0.874–0.920)	0.969 (0.957–0.979)	0.449 (0.378–0.521)	0.905 (0.886–0.921)	0.727 (0.639–0.804)	0.936	0.495	0.085	0.13	0.654
	GBM	0.914 (0.893–0.935)	0.982 (0.972–0.989)	0.439 (0.368–0.511)	0.905 (0.886–0.921)	0.819 (0.732–0.887)	0.942	0.519	0.077	0.152	0.678
	EN	0.908 (0.886–0.930)	0.969 (0.957–0.979)	0.505 (0.433–0.577)	0.914 (0.896–0.93)	0.750 (0.667–0.821)	0.941	0.547	0.078	0.09	0.673
	LR	0.900 (0.878–0.923)	0.968 (0.956–0.978)	0.500 (0.428–0.572)	0.913 (0.895–0.929)	0.742 (0.659–0.815)	0.940	0.540	0.080	0.143	0.657
Comparisons	χ^2^	1.500	4.213	1.500	1.371	2.833	4	4	4		
	*p*	0.827	0.378	0.827	0.849	0.586	0.406	0.406	0.406		
Validation	NN	0.852 (0.804–0.900)	0.969 (0.949–0.983)	0.427 (0.318–0.541)	0.904 (0.874–0.929)	0.714 (0.567–0.834)	0.935	0.474	0.087	0.289	0.564
	SVM	0.851 (0.804–0.898)	0.974 (0.954–0.986)	0.366 (0.262–0.480)	0.895 (0.865–0.921)	0.714 (0.554–0.843)	0.933	0.424	0.089	0.147	0.556
	GBM	0.849 (0.800–0.898)	0.974 (0.954–0.986)	0.341 (0.240–0.454)	0.892 (0.861–0.917)	0.700 (0.535–0.834)	0.931	0.399	0.089	0.216	0.587
	EN	0.851 (0.802–0.899)	0.965 (0.944–0.980)	0.439 (0.330–0.553)	0.905 (0.876, 0.930)	0.692 (0.549–0.813)	0.934	0.475	0.088	0.216	0.571
	LR	0.856 (0.809–0.903)	0.967 (0.946–0.981)	0.402 (0.296–0.517)	0.900 (0.870–0.925)	0.688 (0.537–0.813)	0.932	0.445	0.086	0.227	0.573
Comparisons	χ^2^	1.290	1.433	1.500	1.500	1.089	4	4	4		
	*p*	0.863	0.838	0.827	0.827	0.896	0.406	0.406	0.406		
Cumulative cohort	CORE-COVID-19	0.880 (0.858–0.901)	0.904 (0.889–0.919)	0.669 (0.610–0.724)	0.937 (0.924–0.949)	0.562 (0.507–0.616)	0.921	0.532	0.156	8	0.593
	ISARIC-4C	0.751 (0.720–0.781)	0.794 (0.773–0.814)	0.565 (0.504–0.624)	0.909 (0.892–0.924)	0.334 (0.291–0.379)	0.847	0.28	0.214	12.6	0.359
	CURB-65	0.735 (0.705–0.765)	0.936 (0.923–0.948)	0.295 (0.242–0.352)	0.879 (0.862–0.894)	0.458 (0.384–0.534)	0.907	0.27	0.133	2	0.374
	qSOFA	0.676 (0.644–0.707)	0.967 (0.957–0.975)	0.209 (0.162–0.261)	0.870 (0.853–0.885)	0.537 (0.438–0.633)	0.916	0.234	0.135	1	0.268
	MEWS	0.674 (0.640–0.708)	0.850 (0.831–0.867)	0.378 (0.320–0.438)	0.882 (0.864–0.898)	0.315 (0.266–0.368)	0.865	0.21	0.147	3	0.258

AUC, area under the receiver operating characteristic curve; CI, confidence interval; CORE-COVID-19, Collaboration for Risk Evaluation; COVID-19; CURB-65 score based on confusion, urea, respiratory rate, blood pressure, and age ≥ 65 years; EN, ensemble model; GBM, gradient boosting machine; ISARIC-4C, International Severe Acute Respiratory and emerging Infections Consortium Coronavirus Clinical Characterization Consortium; LR, logistic regression; MEWS, modified early warning score; NN, neural network; qSOFA, quick sequential organ failure assessment; PPV, positive predictive value; NPV, negative predictive value; SVM, support vector machine

*F1 score = 2 × (positive predictive value × sensitivity)/ (positive predictive value + sensitivity); Ranges between 0 and 1, higher the value better the performance: score 0.8–0.9 indicates good and > 0.9 represent very good performance

^**†**^**Kappa = **A measure of the performance of a classification model controlling for the accuracy; score < 0 is indicates no agreement, 0–0.20 as slight, 0.21–0.40 as fair, 0.41–0.60 as moderate, 0.61–0.80 as substantial, and 0.81–1 as almost perfect agreement

^**¶**^**Brier score = **mean squared difference between observed and predicted outcome, a measure of calibration, ranges from 0 to 1 with 0 representing the best and 1 represent worst calibration

**Youden index = sensitivity (%) + specificity (%) – 100; ranges from 0 to 1 with 1 representing perfect test

### CORE-COVID-19 risk prediction model

**Fig. 4:**
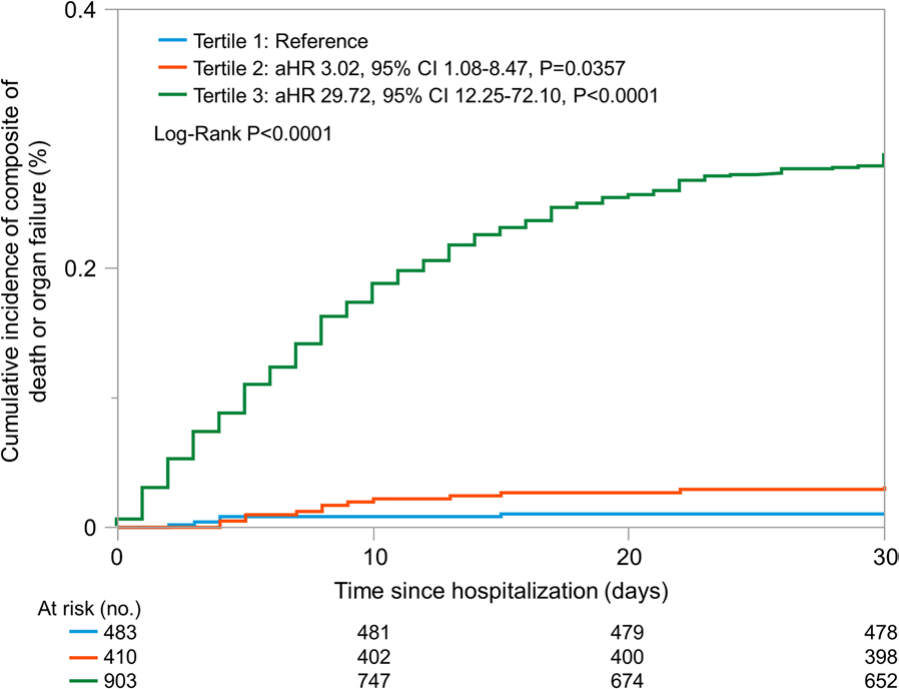
Kaplan-Meir estimates for cumulative incidence of the composite of death or organ failure by the tertiles of COVID-19 organ failure CORE-COVID-19 scores: low, intermediate, and high-risk. In cumulative cohort of 1794 hospitalized COVID-19 patients, 42.5% composite events occurred in highest compared with 7.9% in the intermediate and 1.4% in the lowest tertile. Hazard ratios and 95% confidence intervals were adjusted to demographics. Abbreviations. aHR, adjusted hazard ratio; CI confidence interval

Six variables with the greatest contribution to the model were fitted to develop the CORE-COVID-19 model, with estimated scores ranging from 0 to 16 points to predict the composite outcome. The score assigned to each predictor variable and their weighting in the CORE-COVID-19 model are described in Table 3. To predict the composite outcome, the CORE-COVID-19 model achieved an AUC of 0.880 (95% CI 0.858–0.901). With a cutoff at 8 points, the CORE-COVID-19 model had 90% sensitivity (95% CI 0.889–0.919), 67% specificity (95% CI 0.610–0.724), 94% PPV (95% CI 0.924–0.949), 56% NPV (95% CI 0.507–0.616) with a high F1 score of 0.921, low Brier score of 0.156, and Youden Index of 0.593 for predicting composite outcomes. Additional file 1: Table S3 illustrates the ORs with 95% CIs for each selected variable included in the CORE-COVID-19 model. The CORE-COVID-19 scores were stratified into tertiles (0–4, 5–7, and ≥ 8) for clinical use. After multivariable adjustment for age, sex, and race, patients in the highest tertile (tertile 3) had a 30-fold [hazard ratio (HR) 29.7; 95% CI 12.3–72.1, *P* < 0.0001] and tenfold (HR 9.8, 95% CI 5.6–17.2) higher risk for the composite outcome than those in the lowest and middle tertiles, respectively. Patients with the composite outcome had a median score of 10, compared to 5 in those with no composite outcome (W = 50,530.5, *P* < 0.0001). These findings imply that with a cutoff at 8 points, the CORE-COVID-19 model correctly classified 88% of patients who potentially progressed to death or organ failure by day 30. The Kaplan–Meier curves are illustrated in Fig. 4.

**Table 3:** CORE-COVID-19 score for the composite of intubation, intravenous administration vasopressors, or death within 30-days of hospitalization for COVID-19

Predictor variable	Status	Score
Respiratory failure	No	0
	Yes	4
Admission to critical care unit	No	0
	Yes	3
Exposure to psychoactive medications	No	0
	Yes	2
Hypotension	No	0
	Yes	2
Blood urea nitrogen, mg/dL	< 12	0
	12–30	2
	≥ 31	3
Platelet count, ×10^9^/L	< 135	2
	135–371	1
	≥ 372	0

Score for each variable was derived from logistic regression model

### Comparisons

There were no significant differences between ML models interm of performance metrics (Table 2; Figs. 2A, B, and 3). Notably, the EM provided no additional improvement in discrimination over NN, SVM, GBM, and LR classifier in predicting the composite outcome. However, each ML algorithm and CORE-COVID-19 model outperformed ISARIC-4C, CURB-65, qSOFA, and MEWS in predicting the composite outcomes (Table 2; Fig. 2C). The performance of ISARIC-4C (AUC 0.710) was comparable to that of CURB-65 (AUC 728, *P* = 0.205), qSOFA (AUC 0.678, *P* = 0.124) and MEWS (AUC 0.671, *P* = 0.075) in the study cohort (Table 2; Fig. 2C).

### DCA

DCA results were similar across ML models in the development and validation datasets (Fig. 2D and E). The ML models achieved the best net benefit, approximately 0.07–0.08 when the threshold probability approached the minimum in the development dataset. The maximum net benefit for the CORE-COVID-19 model was at the 0.1 cutoff and continued to reveal net benefit at above 55% of threshold probability which was higher than that of ISARIC-4C, CURB-65, qSOFA, and MEWS (Fig. 2F).

### Check lists

STROBE check list is provided in Additional file 1: Table S5 and TRIPOD check lis in Additional file 1: Table S6.

## Discussion

### Principal findings

Using artificial intelligence approaches, we developed four independent ML models, an EM, and a point-based CORE-COVID-19 risk prediction tool with discrimination and net clinical benefits analysis. The results demonstrated that the ML models and the CORE-COVID-19 model were consistently superior to four existing risk prediction tools for predicting the 30-day composite of death or organ failure in patients hospitalized for first-ever COVID-19 with a broader clinical spectrum. Notably, the EM did not confer any additional benefit. Instead, the improved performances of the de novo models were likely from the feature selection process capturing high-dimensional non-linear interactions, and rigorous ML training and tuning, which might not have been possible with standard statistical methods.

The feature selection process identified the six most predictive variables from multiple domains from a total of 92 potential candidate predictors. The six selected variables were used to train ML models and to develop a new 16-point-based CORE-COVID-19 model. Of the six variables, admitting service (admission to ICU, internal medicine, or other services) was a hospital level characteristic. The Mayo Clinic with its 16 hospitals across four states is a highly integrated and closely regulated healthcare system in the United States. The clinical practice across the Mayo Clinic hospitals including admission to ICU is rather homogenous and all hospitals were continuously and remotely monitored by enhance ICU services. However, subtle differences in practice of admitting patients to the ICU across the differences multiple sites cannot be excluded. The CORE-COVID-19 model with an AUC of 0.880 accurately classified hospitalized COVID-19 patients into low-, intermediate-, and high-risk tertiles for the composite outcome. The CORE-COVID-19 model consistently outperformed ISARIC-4C, CURB-65, qSOFA, and MEWS in outcome prediction. Our findings imply that compared with existing prediction tools; the CORE-COVID-19 model can miss 12%-19% fewer patients at risk of a composite outcome. Furthermore, in the DCA, the CORE-COVID-19 model attained a higher net benefit across a range of thresholds than ISARIC-4C, CURB-65, qSOFA, or MEWS risk scores.

Compared with the ML-derived CORE-COVID-19 model, the modest performance of existing tools could lead to underestimation of the risk, consequent inappropriate interventions, and sub-optimal outcomes. In contrast, the CORE-COVID-19 model improved the precision classification between COVID-19 patients with and without the composite outcome. Notably, the identified predictor variables provided potential insights into disease progression or death and probably accounted for the greater discriminatory ability of the CORE-COVID-19 model in our study.

### Clinical perspective

*Comparison with previously identified predictors.* We identified respiratory failure [53], hypotension, elevated BUN [19], low platelet count [54, 55], admission to ICU [56, 57], and exposure to antipsychotic medication [58] in the hospital as the most predictive variables, all of which were recognized for their respective association with mortality in COVID-19 or other acute conditions. Importantly, the CORE-COVID-19 model shared few predictor variables with ISARIC-4C [44], CURB-65 [19], qSOFA [45], and MEWS [46]. The CORE-COVID-19 is the first prediction model to use a combination of these variables and their respective weightings to predict the outcome. A notable finding of our study was that the risk of progression to composite outcome was strongly associated with disease-specific and hospital-level characteristics as opposed to widely recognized socio-demographics and comorbidities, and certain other laboratory markers, which is supported by a few previous reports that suggested that COVID-19 disease progression was independent of patient-level characteristics[44, 59–63]. We could not identify a few of the most frequently reported prognostic markers included in many risk-stratification scores for COVID-19 such as sex, lymphocyte count, and inflammatory markers. These discordant results were attributed to differences in the study population, study time-frame [62], completeness of data collection [59, 64], distribution of demographics [60], comorbidities [65], geographic sites [66], and class imbalance. Our study’s 30-day composite outcome of death or organ failure was 15%, considerably lower than mortality alone (17–32%) as reported in other regions [44, 61, 67, 68].

*Comparison with existing risk prediction tools.* The discriminatory performances of ISARIC-4C (AUC 0.751 vs 0.767), qSOFA (0.676 vs 0.63) [13] and MEWS (0.674 vs 0.63 [13] in our study were similar to the estimates in the original development and validation studies. In a previous comparative analysis, ML models consistently outperformed CURB-65 [69–71], qSOFA [70, 71] and MEWS [71, 72], which is consistent with our findings.

*Comparisons with previous ML modeling studies.* Studies that described ML prognostic models in patients with COVID-19 have yielded mixed results [4, 73]. Although previously identified ML models achieved modest to excellent discriminatory performance, the studies were at high-risk of bias when assessed using “Prediction model Risk Of Bias Assessment Tool (PROBAST)” [74] [4, 73]. Whereas single-center studies with a small sample size are at high risk for class imbalance, larger studies with pooled data from multiple participating centers are subject to bias related to between-center differences in practice, EHR quality, distribution of comorbidities and other patient characteristics, and treatment pattern [59, 75, 76]. Most ML models for COVID-19 outcomes were developed early during the pandemic, when treatment has rapidly evolved, resulting in time bias [4, 59, 73, 76]. These models may not provide a valid prediction for decision-making in an individual patient, regardless of their accuracy in discrimination and calibration at the population level [75]. In our stud, although drawn from multiple centers across geographically dispersed states, the study population, EHR quality, distribution of demographics and comorbidities, hospital-level care, and treatment patterns were consistent across the integrated Mayo Health System in the United States. These advantages support translation of our findings to bedside clinical practice.

### Clinical implications

Although, the ML algorithms in developing risk prediction model were complex, the six variables that were identified are routinely available. The data collected at the bedside can be analyzed by the point-based, CORE-COVID-19 model to stratify hospitalized COVID-19 patients in to low, intermediate, or high-risk categories for critical organ failure or death at 30 days. The CORE-COVID-19 tool was primarily developed for identifying patients at increased risk for progression to composite of endotracheal intubation, intravenous vasopressor administration, or death. By providing enhanced support for clinical decision-making and allowing the early implementation of appropriate interventions, the CORE-COVID-19 model can potentially lead to lower morbidity and mortality among patients hospitalized for COVID-19.

### Research implications

Our findings warrant further validation in separate datasets with a more heterogenous COVID-19 population, followed by a prospective evaluation of whether the early identification of at-risk patients can improve outcomes. Moreover, as the COVID-19 pandemic continues to evolve with the periodic emergence of SARS-CoV-2 variants with variable transmissibility and disease severity, new data may become available for real-time retraining of ML algorithms for up-to-date risk stratification and support clinical decision making.

### Strengths and limitations

The major strengths were as follow: (1) a broad array of candidate predictors from multiple domains and large well characterized laboratory confirmed cohort of COVID-19 patients; (2) the cohort representative of geographically dispersed regions in the United States; (3) The data collection was nearly complete with minimal variations in data recording and fewer missing data points than in previous studies ensuring robust and transportable findings [59]; (4) The results of the study are likely to enhance the generalizability of the findings and reduce spectrum bias [77]; (5) rigorous ML and data analytics were implemented including feature selection, model development, and calibration; (6) to assess clinical utility of individual models, we compared the de novo models with existing and widely used prognostic tolls as exemplars and conducted DCA analysis for each model to estimate the net benefit across different thresholds (7) the results were displayed in visual graphics for easy understanding of clinical audience, and the report complied with TRIPOD and other recently developed guidelines. Therefore, the present study overcomes many limitations of previously developed models in patients hospitalized for COVID-19. The major limitations of the study were as follow. The study was conducted in the pre-vaccination era before the emergence of delta or omicron variants in the United States. Therefore, the result may be different in contemporary patient populations. The study population was predominantly Caucasians, reflecting the composition of the Mayo Clinic catchment areas. The ML models were not fully automated as the investigators retained the selection of candidate predictors for training.

## Conclusions

The CORE-COVID-19 classifier, based on six clinical variables selected from 92 priori variables through an artificial intelligence approach, accurately assigned 88% of patients who potentially progressed to composite events at 30 days, improving existing risk prediction models based on conventional statistics. These findings indicate that CORE-COVID-19 can be used at the bedside to guide clinical decision-making and improve clinical outcomes.

## Supplementary Information

**Additional file 1. Fig S1.** STROBE flow-diagram. **Table S1.** Machine learning models. **Table S2.** Description of existing risk prediction tools. **Table S3.** Risk prediction models and estimated scores. **Table S4.** Characteristics of study population by the development and validation cohorts. **Panel 1.** Methods, additional description. **Table S5.** STROBE checklist. **Table S6.** TRIPOD checklist.

## Data Availability

The data are not publicly available due to privacy of research participant. The data that support the findings of the present study will be available on specific request from the corresponding author.
