# Supplementary material for: An artificial intelligence approach for predicting death or organ failure after hospitalization for COVID-19: development of a novel risk prediction tool and comparisons with ISARIC-4C, CURB-65, qSOFA, and MEWS scoring systems
Source: Respir Res. 2023 Mar 13;24:79. doi: 10.1186/s12931-023-02386-6 (PMC10010216; doi:10.1186/s12931-023-02386-6)
Supplement: Supplementary file 1 — Additional file 1. Fig S1. STROBE flow-diagram. Table S1. Machine learning models. Table S2. Description of existing risk prediction tools. Table S3. Risk prediction models and estimated scores. Table S4. Characteristics of study population by the development and validation cohorts. Panel 1. Methods, additional description. Table S5. STROBE checklist. Table S6. TRIPOD checklist. [file 12931_2023_2386_MOESM1_ESM.pdf]

Supplement

Title

An artificial intelligence approach for predicting death or organ failure after hospitalization for COVID-19: development of a novel risk stratification tool and comparisons with ASARIC-4C, CURB-65, qSOFA, MEWS scoring systems.

Page no.

Supplement Figure 1. STROBE flow-diagram…………………………………………………………………………………….. 2
Supplement Table 1. Machine learning models……………………………………………………………………………… 3
Supplement Table 2. Description of existing risk prediction tools………………………………………………………. 4
Supplement Table 3. Risk prediction models and estimated scores……………………………………………………. 5
Supplement Table 4. Characteristics of study population by the development and validation cohorts………. 7
Supplement Panel 1. Methods, additional description. ……………………………………………………………………… 9
Supplement Table 5. STROBE checklist…………………………………………………………………………………………. 11
Supplement Table 6. TRIPOD checklist…………………………………………………………………………………………. 14
References……… 16

Supplement Figure 1. Strengthening the Reporting of Observational Studies in Epidemiology (STROBE) flow diagram for study cohort selection.

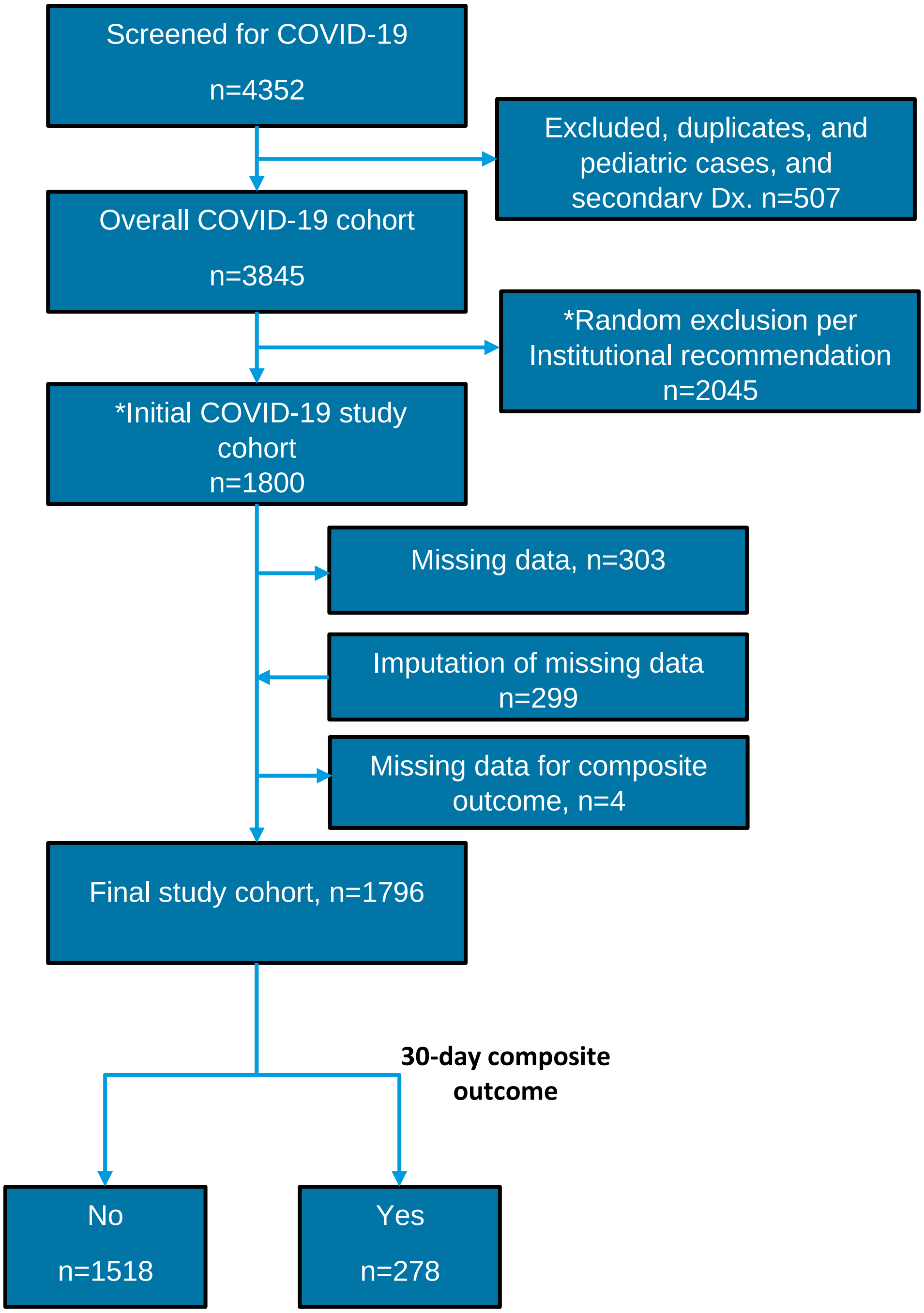

*The group included in the study (n=1800) was comparable to the group randomly excluded (n=1415) in accordance with institutional recommendation for data sharing.

Supplement Table 1. Machine learning models

Model	Description	Advantageous	Disadvantageous
**Gradient Boost Machine (GBM)**	Builds an ensemble of sequential weak models. Each successive model learns, reduces the misclassification errors, and improve on the prior models.	Handle large data, minimize bias error, performs well on unprocessed and missing data, provides high accuracy.	Long computational time, potential for overfitting, less understandable.
**Neural network (NN)**	A computational model to discover relationships within complex dataset and to predict outcome for individual patient functioning through interconnected nodes or artificial neurons in a layer that are fully connected to all other artificial neurons in next layers, simulating human brain	Ability to learn from the input data, perform multiple functions simultaneously, and store information on the network.	Long computational time, less interpretable output.
**Support vector machine (SVM)**	A supervised machine learning model for classification and regression analysis	Performs well with high-dimensional, unstructured data with clear margin of separation between classes and lower risk of over-fitting	Not suitable for large dataset with overlapping classes. Difficult fine-tuning of hyper-parameters.

Supplement Table 2. Description of existing risk prediction tools

Risk prediction score	Description
**ISARIC-4C**	A 21point score based on 8 candidate predictors was developed in a large cohort of COVID-19 patients from the United Kingdom to predict in-hospital mortality [1] and successfully validated in external datasets [2,3].
**CURB-65**	A 6-point score with 1 point each for confusion, BUN (≥20), respiratory rate ≥30/min, low blood pressure (systolic <90 or diastolic <60), and age was originally developed to estimate 30-day mortality in patients with community acquired pneumonia [4] and widely used in clinical practice for risk stratification. Several clinical trials attempted to validate CURB-65 in COVID-19 population with mixed results [5,6].
**qSOFA.**	A 3-point (1 point each for systolic blood pressure ≤100 mmHg, respiratory rate ≥ 22/min, or altered mental state) score was introduced in 2016 for simple risk stratification of patients with sepsis from diverse conditions outside of the intensive care unit and validated in external datasets [7]and in COVID-19 patients [5].
**MEWS**	A simple bed-side tool based on physiological measures designed to identify undifferentiated hospitalized patients at risk for clinical deterioration and is widely used by nursing staff to alert physicians in everyday hospital practice [8]and previously validated in patients with COVID-19 [9].

Abbreviations:
ISARIC-4C, International Severe Acute Respiratory and emerging Infections Consortium Coronavirus Clinical Characterization Consortium
CURB-65, confusion, urea, respiratory rate, blood pressure, and age ≥ 65 years
qSOFA, quick sequential organ failure assessment
MEWS, modified early warning score

Supplement Table 3. Risk prediction models and estimated scores

Risk prediction models	Score
**CURB-65**	
**Confusion/metabolic encephalopathy**	1
**BUN >19 mg/dl**	1
**Respiratory rate >30**	1
**SBP <90 or DBP <60**	1
**Age ≥ 65**	1
**qSOFA**	
**Altered mental status/metabolic encephalopathy**	1
**Respiratory rate ≥22**	1
**SBP ≤100**	1
**MEWS score**	
**SBP**	
≤ 70 mmHg	3
71 – 80 mmHg	2
81 – 100 mmHg	1
101 – 199 mmHg	0
≥ 200 mmHg	2
**Heart rate**	
<40	2
41-50	1
51 - 100	0
101 - 110	1
111 - 129	2
≥130	3
**Respiratory rate**	
< 9	2
9-14	0
15-20	1
21-29	2
≥30	3
**Temperature**	
<35	2
35-38.4	0
≥38.5	2
**Altered mental state/metabolic encephalopathy**	
no	0
yes	2
**4C mortality score**	
**Age**	
<50	0
50-59	2
60-69	4
70-79	6
≥80	7
**Sex**	
Female	0
Male	1

**Comorbidities, number**	
0	0
1	1
≥2	2
**Respiratory rate**	
<20	0
20-29	1
≥30	2
**Peripheral oxygen saturation (SpO2)**	
≥92%	0
<92%	2
**Metabolic encephalopathy**	
No	0
Yes	2
**BUN**	
<19.6 mg/dl	0
19.6-39.2 mg/dl	1
>39.2	3
**C-reactive protein**	
<50 mg/L	0
50-99 mg/L	1
≥ 100	2

Supplement Table 4. Characteristic of study population by development and validation cohorts.

Category	Variable	Development cohort, n = 1258	Validation cohort n = 538	P value
**Demographics**	Age	65.9 (14.9)	67.1 (14.7)	0.1175
	Female	537 (43)	219 (41)	0.4361
	White	1029 (82)	458 (85)	0.0864
**Social indicators**	Married	764 (61)	311 (58)	0.2468
	Current smoker	62 (5)	24 (5)	0.6708
	Ever smoker	492 (39)	204 (38)	0.6350
	Substance use disorder	42 (3)	18 (3)	0.9939
**Anthropometric measure**	Body mass index	31.1 (7.6)	31.2 (8.2)	0.9416
**Admission source**	Home	1007 (80)	428 (80)	0.8110
	Clinic	41 (3)	24 (5)	0.2116
	Acute care	142 (11)	50 (10)	0.4362
	Nursing home	68 (5)	32 (6)	0.6460
**Admitting service**	Intensive care unit	141 (11)	46 (8)	0.0911
	Internal medicine	774 (62)	335 (62)	0.7670
	Other service	343 (27)	157 (29)	0.4065
**Comorbid conditions**	Anemia	304 (24)	124 (23)	0.6108
	Arthritis	138 (11)	67 (12)	0.3650
	Atrial fibrillation	280 (22)	124 (23)	0.7131
	Asthma	85 (7)	38 (7)	0.8138
	Bone marrow disease	65 (5)	31 (6)	0.6075
	Bone marrow/stem cell transplant	7 (0.6)	9 (1.7)	0.0211
	Coronary artery disease	118 (9)	46 (9)	0.5760
	Cancer, active	78 (6)	26 (5)	0.2557
	Cancer with metastasis	33 (3)	8 (2)	0.1397
	Chronic kidney disease	283 (22)	128 (24)	0.5493
	Chronic obstructive pulmonary disease	190 (15)	85 (16)	0.7075
	Depression	236 (19)	105 (20)	0.7080
	Dementia	21 (2)	21 (3)	0.0041
	Diabetes mellitus	493 (40)	197 (37)	0.3046
	Diabetes with complications	271 (21)	123 (23)	0.5357
	Heart failure	189 (15)	116 (21)	0.0007
	Human immuno-deficiency virus	3 (0.2)	0	0.2569
	Hyperlipidemia	651(52)	287 (53)	0.5349
	Hypertension	837 (67)	357 (66)	0.9419
	Immunodeficiency	57 (5)	29 (5)	0.4346
	Liver disease	43 (3)	7 (1)	0.0125
	Malnutrition	101 (8)	51 (9)	0.3116
	Obstructive sleep apnea	296 (24)	129 (24)	0.8379
	Osteoporosis	78 (6)	42 (8)	0.2117
	Other lung conditions	70 (6)	30 (6)	0.9920
	Other neurological conditions	43 (3)	20 (4)	0.7521
	Other psychiatric conditions	96 (8)	41 (8)	0.9940
	Peripheral artery disease	3 (0.2)	5 (1)	0.0440
	Solid organ transplant	99 (5)	26 (5)	0.8962
	Stroke	51 (4)	31 (6)	0.1122

	Venous thrombo-embolism	143 (11)	54 (10)	0.4087
**Vitals**	Heart rate	90 (18)	89 (17)	0.4901
	Respiratory rate	22 (5)	22 (6)	0.4302
	Systolic blood pressure	131 (20)	130 (19)	0.2581
	Diastolic blood pressure	77 (12)	76 (12)	0.3097
	Temperature	37.1 (0.7)	37 (0.6)	0.9520
	SpO2, 94% – 100%	766 (61)	331 (62)	0.8008
	90% – 93%	377 (30)	156 (29)	0.6797
	<90%	115 (9)	51 (9)	0.8207
**Laboratory measures**	Hemoglobin (g/dL)	13 (2)	13 (2)	0.8772
	White blood cell count	6.4 (4.6, 8.9)	6.5 (5.0, 9.0)	0.2999
	Neutrophils	4.8 (3.3, 7.2)	4.8 (3.4, 7.1)	0.5513
	Lymphocytes	0.8 (0.6, 1.1)	0.8 (0.6, 1.2)	0.6050
	Platelets	206 (87)	209 (88)	0.5581
	Sodium	135.1 (4.5)	135.1 (5.4)	0.9425
	Bicarbonate	23.4 (3.7)	23.5 (3.6)	0.7014
	Anion gap	12.7 (3.0)	12.5 (2.9)	0.2787
	Blood urea nitrogen	18 (13, 27)	18 (13, 28)	0.8400
	Creatinine	1 (0.8, 1.3)	1.0 (0.8, 1.4)	0.3140
	Albumin	3.6 (0.5)	3.5 (0.5)	0.2394
	Alanine transaminase	29 (14, 47)	28 (19, 44)	0.2799
	Aspart transaminase	38 (30, 58)	39 (29, 58)	0.4586
	Bilirubin	0.5 (0.3, 0.6)	0.5 (0.3, 0.7)	0.6074
	Glucose	141 (61)	137 (49)	0.1609
	C-reactive protein	63.8 (26.6, 121.2)	72.1 (32.9, 190.5)	0.0597
**ECG measure**	QTc interval	449 (31)	451 (31)	0.4207
**In-hospital complications**	Hypothermia	12 (0.9)	3 (0.6)	0.3980
	Hypotension	185 (15)	94 (17)	0.1382
	Myocardial infarction	19 (1)	11 (2)	0.4184
	Cardiac arrest	16 (1)	6 (1)	0.7822
	Respiratory failure	717 (57)	297 (55)	0.4832
	Pulmonary edema	30 (2)	10 (2)	0.4890
	Pulmonary embolism	113 (9)	39 (7)	0.2267
	Secondary pneumonia	169 (13)	67 (12)	0.5732
	GI complications	40 (3)	16 (3)	0.8183
	Electrolyte abnormality	772 (61)	348 (65)	0.1839
	Disseminated intravascular coagulation	8 (0.6)	0	0.0638
	Encephalopathy	90 (7)	38 (7)	0.5732
**Drugs**	Remdesivir	145 (12)	62 (12)	0.9990
	Tocilizumab	20 (2)	10 (2)	0.6838
	Dexamethasone	297 (24)	124 (23)	0.7973
	Hydroxychloroquine	29 (3)	7 (1)	0.1643
	Aspirin	440 (35)	205 (38)	0.2056
	Statin	461 (37)	208 (39)	0.4182
	ACEI/ARBs	351 (28)	135 (25)	0.2198
	Anti-psychotic medication	99 (8)	48 (9)	0.4562
	SSRI	161 (13)	69 (13)	0.9874

Abbreviations: ACEI/ARBs, angiotensin converting enzyme inhibitor/Angiotensin II receptor blockers; SSRI, selective serotonin receptor inhibitors

Panel 1. Methods

Data pre-processing

The missing values for continuous variable were imputed by bagged trees method and for dichotomous variables by the mode value [10,11]. The continuous variables were further transformed by the Yeo-Johnson transformation to reduce skewness, and then centered and scaled. The pre-processed data were randomly split [12] into training (70%) and validation (30%) sets for model development and internal validation. See Appendix methods section for further details for data pre-processing.

Feature engineering (variable selection)

Variable selection was performed to eliminate potentially unrelated variables and to enhance performance of prediction model [13]. We used recursive feature elimination (RFE) with sequential backward variable selection method to identify a set of variables that are most important for prediction [14,15]. Training data set comprised of 1258 patients each with 98 candidate predictors and one composite outcome. The RFE procedure was performed with logistic regression, naïve Bayes, and random forest algorithms. Each model was run with 5-repeats of 10-fold cross-validation. we calculated the level of importance of variables in the chosen model [16]. See Appendix methods section for further details of feature engineering.

Machine learning analytic approach

Machine learning-based models. Firstly, we fitted a logistic regression model as the reference model and then used predictor variables to constructed 4 independent ML algorithms to predict the probability of composite outcome In the development set (N = 1258, 70%): 1) artificial neural network (NN)[17], 2) support vector machine (SVM) [18], 3) gradient boosting (GBM) [19], and 4) Logistic regression (LR) [20]. We reported summary of ML models in Supplemental Table.

Model development. Each algorithm was tuned via random grid search from 300 candidate parameter or parameters combination, with 10-fold cross-validation, repeated 5 times [21,22].

Ensemble model (EM). We combined the results NN, SVM, and GBM models to generated an ensemble ML model, a single ML-model that combined multiple classification models using linear regression [23] and computed integrated discrimination improvement to estimate the added value of ensemble model [24].

Logistic regression (LR). run on raw data to regress composite outcome on the selected variables to compute estimates and odds ratios with 95% confidence intervals (CI).

Development of point-based CORE-COVID-19 model

A point-based scoring system was developed based on the work of Xie and colleagues [25] to risk stratify the patients for the composite outcome using the selected features from the RFE processing. The continuous independent variables were converted into categorical ones based on five quantiles i.e., 0.05, 0.2, 0.8, 0.95, and 1 [26]. Logistic regression was run for score weighting for each variable's categories in the training data set.

The cut-off values of the continuous variables were fine-tuned based on the first weighting results. Performance metrics were obtained from the validation dataset after fine-tuning. The total score was set at 16 for an easy manual calculation.

External validation and evaluation of existing risk-prediction tools

We conducted a systematic search of PubMed, Embase, Web of Science, Cochrane library, CINAHL databases to identify 4 risk prediction scores (ISARIC-4C, CURB-65 score, qSOFA, and MEWS) that are widely used to predict in-hospital mortality in diverse acute conditions and feasible in the current dataset for validation. The ISARIC-4C was originally developed in hospitalized COVID-19 population in the United Kingdom. Although, CURB-65, qSOFA, and MEWS were developed in non-COVID-19 population, they share similar characteristics and their prognostic implications in COVID-19 has recently been explored. The ISARIC-4C, CURB-65, qSOFA, and MEWS scores were calculated for each patient. The dichotomous Glasgow coma scale (15 vs < 15) was replaced by presence or absence of metabolic encephalopathy and the unit of BUN in mg/dl was multiplied by a conversion factor of 0.3571 to convert to mmol/L for estimating ISARIC-4C score [27]. The ISARIC-4C, CURB-65, qSOFA, and MEWS scores, each, externally validated and recalibrated. For each existing scoring system, the total score was rescaled to range from 0 to 1. The cut-off of rescaled score was identified using Youden index based on rescale score and true classes of composite outcome. The cutoff for total score was calculated by multiplying the rescaled cutoff value with the total score. The binary class prediction was determined by categorizing patients'

total score according to cut-off value. The rescaled scores and predicted classes were used for the evaluation of model performance. Scores were estimated for each model against their 30-day validated outcome in patients with COVID-19. Original scores were fitted to reconstruct each risk prediction model in cumulative COVID-19 cohort.

Statistical analysis

General. We reported, the mean and standard deviation (SD) for normally distributed variables, median and interquartile range (IQR) for non-normally distributed variables, and the number and proportion for categorical variables. We used Student *t* test for normally distributed data, Kruskal-Wallis for non-normally distributed data, Pearson $\chi 2$ test for categorical variables for univariate analyses as appropriate. The statistical significance was adjusted to P <0.0005 to account for multiple comparisons of baseline variables using Bonferroni's method.

Standard performance metrics. Performances of ML models were evaluated both in the development and validation datasets whereas performances of ISARIC-4C, CURB-65, qSOFA, and MEWS were assessed in the cumulative cohort. Discrimination was quantified using area AUC. ROC curves were generated for each ML algorithm CORE-COVID-19 model, and the existing prediction scoring systems. Because AUC does not account for outcome prevalence, we reported sensitivity, specificity, positive predictive value (PPV), negative predictive value (NPV), and accuracy at a specific risk threshold. Performance of the model was rated using F1 score and Kappa statistics. Performance metrics were compared by Kruskal-Wallis test across the models whereas goodness-of-fit was assessed by Hosmer-Lemeshow test [28]. A model was considered significantly different if its 95% CI excepted the point estimate of other model and vice versa and significance level <0.05.

Calibration. Agreement between probability of prediction and actual observation was estimated for each model separately for the development and validation sets [29]. For each model, calibration was assessed using Brier score, Hosmer-Lemeshow test, and calibration plots.

Decision curve analysis (DCA). We performed DCA to determine the net benefit relative to harm in predicting the composite outcome [30] DCA accounts for the tradeoff between harms and benefits across a range of thresholds for the model to ascertain whether to risk stratify the patients using the model [31].

Visual display of Comparative performances. Separate ROC curves and calibration plots were generated in development and validation datasets and DCA plots were created in the cumulative cohort for each model.

Analysis of CORE-COVID-19 model. Logistic regression analysis was conducted to regress study outcome on the selected variables (that eventually constituted risk prediction tool) to compute estimates and odds ratios (OR). CORE-COVID-19 scores were stratified into tertiles of equal sizes to support clinical use. The effect, according to tertile, on composite outcome was analyzed using Kaplan-Meier method and Cox regression model.

Supplement Table 5. STROBE check list

Strengthening The Reporting of Observational studies in Epidemiology (STROBE) Statement

Checklist of items that is included

Section/Topic	Item No	Recommendation	Reported on Page No
Title and abstract	1	(*a*) Indicate the study's design with a commonly used term in the title or the abstract	1
		(*b*) Provide in the abstract an informative and balanced summary of what was done and what was found	2
Introduction			
Background/rationale	2	Explain the scientific background and rationale for the investigation being reported	4, 5
Objectives	3	State specific objectives, including any pre-specified hypotheses	4, 5
Methods			
Study design	4	Present key elements of study design early in the paper	5, 6
Setting	5	Describe the setting, locations, and relevant dates, including periods of recruitment, exposure, follow-up, and data collection	5, 6
Participants	6	(*a*) *Cohort study*—Give the eligibility criteria, and the sources and methods of selection of participants. Describe methods of follow-up<br>*Case-control study*—Give the eligibility criteria, and the sources and methods of case ascertainment and control selection. Give the rationale for the choice of cases and controls<br>*Cross-sectional study*—Give the eligibility criteria, and the sources and methods of selection of participants	5,6
		(*b*) *Cohort study*—For matched studies, give matching criteria and number of exposed and unexposed<br>*Case-control study*—For matched studies, give matching criteria and the number of controls per case	
Variables	7	Clearly define all outcomes, exposures, predictors, potential confounders, and effect modifiers. Give diagnostic criteria, if applicable	6-10
Data sources/measurement	8*	For each variable of interest, give sources of data and details of methods of assessment (measurement). Describe comparability of assessment methods if there is more than one group	6-10
Bias	9	Describe any efforts to address potential sources of bias	6-7
Study size	10	Explain how the study size was arrived at	Not applicable

Quantitative variables	11	Explain how quantitative variables were handled in the analyses. If applicable, describe which groupings were chosen and why	6,7
Statistical methods	12	(*a*) Describe all statistical methods, including those used to control for confounding	6-11
		(*b*) Describe any methods used to examine subgroups and interactions	
		(*c*) Explain how missing data were addressed	6, 7
		(*d*) *Cohort study*—If applicable, explain how loss to follow-up was addressed<br>*Case-control study*—If applicable, explain how matching of cases and controls was addressed<br>*Cross-sectional study*—If applicable, describe analytical methods taking account of sampling strategy	n/a
		(*e*) Describe any sensitivity analyses	n/a

Results			
Participants	13*	(a) Report numbers of individuals at each stage of study—e.g., numbers potentially eligible, examined for eligibility, confirmed eligible, included in the study, completing follow-up, and analysed	11, 12
		(b) Give reasons for non-participation at each stage	
		(c) Consider use of a flow diagram	Supplement Figure 1
Descriptive data	14*	(a) Give characteristics of study participants (e.g., demographic, clinical, social) and information on exposures and potential confounders	11-13
		(b) Indicate number of participants with missing data for each variable of interest	11
		(c) *Cohort study*—Summarise follow-up time (e.g., average and total amount)	10
Outcome data	15*	*Cohort study*—Report numbers of outcome events or summary measures over time	10
		Case-control study—Report numbers in each exposure category, or summary measures of exposure	10
		Cross-sectional study—Report numbers of outcome events or summary measures	10
Main results	16	(*a*) Give unadjusted estimates and, if applicable, confounder-adjusted estimates and their precision (e.g., 95% confidence interval). Make clear which confounders were adjusted for and why they were included	11-14
		(*b*) Report category boundaries when continuous variables were categorized	6, 7
		(*c*) If relevant, consider translating estimates of relative risk into absolute risk for a meaningful time period	
Other analyses	17	Report other analyses done—e.g., analyses of subgroups and interactions, and sensitivity analyses	n/a
Discussion			
Key results	18	Summarize key results with reference to study objectives	11-14

Limitations	19	Discuss limitations of the study, taking into account sources of potential bias or imprecision. Discuss both direction and magnitude of any potential bias	18-19
Interpretation	20	Give a cautious overall interpretation of results considering objectives, limitations, multiplicity of analyses, results from similar studies, and other relevant evidence	14-17
Generalizability	21	Discuss the generalizability (external validity) of the study results	18
Other Information			
Funding	22	Give the source of funding and the role of the funders for the present study and, if applicable, for the original study on which the present article is based	

**Give information separately for cases and controls in case-control studies and, if applicable, for exposed and unexposed groups in cohort and cross-sectional studies.*

Note: An Explanation and Elaboration article discusses each checklist item and gives methodological background and published examples of transparent reporting. The STROBE checklist is best used in conjunction with this article (freely available on the Web sites of PLoS Medicine at http://www.plosmedicine.org

Supplement Table 6. TRIPOD check list

Section/Topic			Checklist Item	Page
**Title and abstract**				
Title	1	D;V	Identify the study as developing and/or validating a multivariable prediction model, the target population, and the outcome to be predicted.	1
Abstract	2	D;V	Provide a summary of objectives, study design, setting, participants, sample size, predictors, outcome, statistical analysis, results, and conclusions.	2
**Introduction**				
Background and objectives	3a	D;V	Explain the medical context (including whether diagnostic or prognostic) and rationale for developing or validating the multivariable prediction model, including references to existing models.	4, 5
	3b	D;V	Specify the objectives, including whether the study describes the development or validation of the model or both.	4, 5
**Methods**				
Source of data	4a	D;V	Describe the study design or source of data (e.g., randomized trial, cohort, or registry data), separately for the development and validation data sets, if applicable.	5, 6
	4b	D;V	Specify the key study dates, including start of accrual; end of accrual; and, if applicable, end of follow-up.	5
Participants	5a	D;V	Specify key elements of the study setting (e.g., primary care, secondary care, general population) including number and location of centres.	5, 6
	5b	D;V	Describe eligibility criteria for participants.	5, 6
	5c	D;V	Give details of treatments received, if relevant.	na
Outcome	6a	D;V	Clearly define the outcome that is predicted by the prediction model, including how and when assessed.	10
	6b	D;V	Report any actions to blind assessment of the outcome to be predicted.	na
Predictors	7a	D;V	Clearly define all predictors used in developing or validating the multivariable prediction model, including how and when they were measured.	12
	7b	D;V	Report any actions to blind assessment of predictors for the outcome and other predictors.	na
Sample size	8	D;V	Explain how the study size was arrived at.	na
Missing data	9	D;V	Describe how missing data were handled (e.g., complete-case analysis, single imputation, multiple imputation) with details of any imputation method.	6, 7
Statistical analysis methods	10a	D	Describe how predictors were handled in the analyses.	6, 12
	10b	D	Specify type of model, all model-building procedures (including any predictor selection), and method for internal validation.	7 - 10
	10c	V	For validation, describe how the predictions were calculated.	10
	10d	D;V	Specify all measures used to assess model performance and, if relevant, to compare multiple models.	10
	10e	V	Describe any model updating (e.g., recalibration) arising from the validation, if done.	9, 10
Risk groups	11	D;V	Provide details on how risk groups were created, if done.	11
Development vs. validation	12	V	For validation, identify any differences from the development data in setting, eligibility criteria, outcome, and predictors.	13
**Results**				
Participants	13a	D;V	Describe the flow of participants through the study, including the number of participants with and without the outcome and, if applicable, a summary of the follow-up time. A diagram may be helpful.	11, 12
	13b	D;V	Describe the characteristics of the participants (basic demographics, clinical features, available predictors), including the number of participants with missing data for predictors and outcome.	11, 12 Table 1, and Suppleme nt Table 4
	13c	V	For validation, show a comparison with the development data of the distribution of important variables (demographics, predictors and outcome).	14
Model development	14a	D	Specify the number of participants and outcome events in each analysis.	11-14
	14b	D	If done, report the unadjusted association between each candidate predictor and outcome.	na
Model specification	15a	D	Present the full prediction model to allow predictions for individuals (i.e., all regression coefficients, and model intercept or baseline survival at a given time point).	13, 14
	15b	D	Explain how to the use the prediction model.	13, 14
Model performance	16	D;V	Report performance measures (with CIs) for the prediction model.	13, 14
Model-updating	17	V	If done, report the results from any model updating (i.e., model specification, model performance).	13, 14
**Discussion**				

Limitations	18	D;V	Discuss any limitations of the study (such as nonrepresentative sample, few events per predictor, missing data).	18, 19
Interpretation	19a	V	For validation, discuss the results with reference to performance in the development data, and any other validation data.	14, 15
	19b	D;V	Give an overall interpretation of the results, considering objectives, limitations, results from similar studies, and other relevant evidence.	16-18
Implications	20	D;V	Discuss the potential clinical use of the model and implications for future research.	17, 18
**Other information**				
Supplementary information	21	D;V	Provide information about the availability of supplementary resources, such as study protocol, Web calculator, and data sets.	19, 20
Funding	22	D;V	Give the source of funding and the role of the funders for the present study.	19

*Items relevant only to the development of a prediction model are denoted by D, items relating solely to a validation of a prediction model are denoted by V, and items relating to both are denoted D;V. We recommend using the TRIPOD Checklist in conjunction with the TRIPOD Explanation and Elaboration document.

References

1. Knight SR, Ho A, Pius R, et al. Risk stratification of patients admitted to hospital with covid-19 using the ISARIC WHO Clinical Characterisation Protocol: development and validation of the 4C Mortality Score. *Bmj* 2020; **370**: m3339.
2. Jones A, Pitre T, Junek M, et al. External validation of the 4C mortality score among COVID-19 patients admitted to hospital in Ontario, Canada: a retrospective study. *Sci Rep* 2021; **11**(1): 18638.
3. Ong SWX, Sutjipto S, Lee PH, et al. Validation of ISARIC 4C mortality and deterioration scores in a mixed vaccination status cohort of hospitalized COVID-19 patients in Singapore. *Clin Infect Dis* 2022.
4. Lim WS, van der Eerden MM, Laing R, et al. Defining community acquired pneumonia severity on presentation to hospital: an international derivation and validation study. *Thorax* 2003; **58**(5): 377-82.
5. Bradley P, Frost F, Tharmaratnam K, Wootton DG. Utility of established prognostic scores in COVID-19 hospital admissions: multicentre prospective evaluation of CURB-65, NEWS2 and qSOFA. *BMJ Open Respir Res* 2020; **7**(1).
6. Elmoheen A, Abdelhafez I, Salem W, et al. External Validation and Recalibration of the CURB-65 and PSI for Predicting 30-Day Mortality and Critical Care Intervention in Multiethnic Patients with COVID-19. *Int J Infect Dis* 2021; **111**: 108-16.
7. Seymour CW, Liu VX, Iwashyna TJ, et al. Assessment of Clinical Criteria for Sepsis: For the Third International Consensus Definitions for Sepsis and Septic Shock (Sepsis-3). *Jama* 2016; **315**(8): 762-74.
8. Subbe CP, Kruger M, Rutherford P, Gemmel L. Validation of a modified Early Warning Score in medical admissions. *Qjm* 2001; **94**(10): 521-6.
9. Aygun H, Eraybar S. The role of emergency department triage early warning score (TREWS) and modified early warning score (MEWS) to predict in-hospital mortality in COVID-19 patients. *Ir J Med Sci* 2021: 1-7.
10. Kuhn M. 3 Pre-Processing. 2022. https://topepo.github.io/caret/pre-processing.html2022).
11. RDocumentation. preProcess: Pre-Processing of Predictors. 2022. https://www.rdocumentation.org/packages/caret/versions/6.0-90/topics/preProcess.
12. RDocumentation. createDataPartition: Data Splitting function. 2022. https://www.rdocumentation.org/packages/caret/versions/6.0-90/topics/createDataPartition.
13. Yang S, Li B, Zhang Y, et al. Selection of features for patient-independent detection of seizure events using scalp EEG signals. *Comput Biol Med* 2020; **119**: 103671.
14. RDocumentation. rfe: Backwards Feature Selection. 2022. https://www.rdocumentation.org/packages/caret/versions/6.0-90/topics/rfe.2022).
15. RDocumentation. rfeControl: Controlling the Feature Selection Algorithms. 2022. https://www.rdocumentation.org/packages/caret/versions/6.0-90/topics/rfeControl2022).
16. RDocumentation. varImp: Calculation of variable importance for regression and classification model. 2022. https://www.rdocumentation.org/packages/caret/versions/6.0-90/topics/varImp2022).
17. RDocumentation. avNNet: Neural Networks Using Model Averaging. 2021. 2022. https://www.rdocumentation.org/packages/caret/versions/6.0-90/topics/avNNet2022).
18. RDocumentation. Kuhn, M. caret/RegressionTests/Code/svmRadial.R. 2017 2022. https://github.com/topepo/caret/blob/master/RegressionTests/Code/svmRadial.R2022).
19. RDocumentation. gbm: Generalized Boosted Regression Modeling (GBM). 2021. 2022. https://www.rdocumentation.org/packages/gbm/versions/2.1.8/topics/gbm.
20. RDocumentation. bayesglm: Bayesian generalized linear models. 2021. 2021. https://www.rdocumentation.org/packages/arm/versions/1.9-3/topics/bayesglm2022).

21. RDocumentation. caretList: Create a list of several train models from the caret package Build a list of train objects suitable for ensembling using the caretEnsemble function. 2021 2021. https://www.rdocumentation.org/packages/caretEnsemble/versions/2.0.1/topics/caretList2022).
22. RDocumentation. trainControl: Control parameters for train. 2021 2021. https://www.rdocumentation.org/packages/caret/versions/6.0-90/topics/trainControl.
23. Gunes H PM. Affect recognition from face and body: early fusion vs. late fusion. https://ieeexplore.ieee.org/document/1571679 (accessed June 2, 2022 2022).
24. Pencina MJ, D'Agostino RB, Sr., D'Agostino RB, Jr., Vasan RS. Evaluating the added predictive ability of a new marker: from area under the ROC curve to reclassification and beyond. *Stat Med* 2008; **27**(2): 157-72; discussion 207-12.
25. Xie F, Chakraborty B, Ong MEH, Goldstein BA, Liu N. AutoScore: A Machine Learning-Based Automatic Clinical Score Generator and Its Application to Mortality Prediction Using Electronic Health Records. *JMIR Med Inform* 2020; **8**(10): e21798.
26. rdrr.io. AutoScore: An Interpretable Machine Learning-Based Automatic Clinical Score Generator. 2022. 2022. https://rdrr.io/cran/AutoScore/2022).
27. Tuchman S, Khademian ZP, Mistry K. Dialysis disequilibrium syndrome occurring during continuous renal replacement therapy. *Clin Kidney J* 2013; **6**(5): 526-9.
28. Kramer AA, Zimmerman JE. Assessing the calibration of mortality benchmarks in critical care: The Hosmer-Lemeshow test revisited. *Crit Care Med* 2007; **35**(9): 2052-6.
29. Van Calster B, McLernon DJ, van Smeden M, Wynants L, Steyerberg EW. Calibration: the Achilles heel of predictive analytics. *BMC Med* 2019; **17**(1): 230.
30. Vickers AJ, Elkin EB. Decision curve analysis: a novel method for evaluating prediction models. *Med Decis Making* 2006; **26**(6): 565-74.
31. Localio AR, Goodman S. Beyond the usual prediction accuracy metrics: reporting results for clinical decision making. *Ann Intern Med* 2012; **157**(4): 294-5.
